# Results of a patient engagement training for health advisors: a study of self-perceived competency enhancements

**DOI:** 10.1186/s40900-025-00711-5

**Published:** 2025-05-23

**Authors:** Eduardo Perez-Guagnelli, Nicole Jardine, Amye Leong, Chris Rao, Michael Cohen, Annabel De Maria

**Affiliations:** 1 PE, Alira Health, Avinguda Josep Tarradellas, 123 (7 th Floor), 08029 Barcelona, Spain; 2 PE, Alira Health, 30 Adelaide Street East (12 th Floor), Toronto, ON M5 C 3G8 Canada; 3 Center for Patient Advocacy Leaders, Healthy Motivation, 883 San Antonio Creek Rd, Santa Barbara, CA 93111 USA; 4 Clinical Operations, Alira Health 1 Grant Street (4 th Floor), Framingham, MA 01702 USA; 5 Center for Patient Advocacy Leaders, MJC HealthSolutions, 4 Upper Flanders Rd, Amherst, NH 03031 USA

**Keywords:** Patient engagement, Healthcare consultant, KSA framework, Product development lifecycle

## Abstract

**Background:**

In recent years, there has been increasing recognition of the importance of patient engagement (PE) in the healthcare industry, especially throughout the product development lifecycle. However, there is limited research on the influence of PE training on the attitudes, knowledge, and skills of health advisors working in the life sciences, who have a substantial effect on decisions made throughout the product lifecycle in the healthcare industry. This study aimed to assess the effectiveness of a patient engagement training course tailored to healthcare consultants, focusing on changes in self-perceived knowledge, skills and attitudes before and after training.

**Methods:**

Eighty healthcare consultants of varying seniority levels from a single company based throughout the U.S. and Europe completed a six-part online training course on patient engagement. The training covered various concepts, and the participants were assessed via a modified evidence-based practice questionnaire before and after the course to measure changes in self-perceived knowledge, skills, and attitudes. The study used paired samples t-tests and bivariate Pearson's correlation analyses to evaluate the differences.

**Results:**

Following the training, the consultants reported significant improvements in their perceived KSAs toward patient engagement (PE). The most substantial increase was observed in knowledge scores, followed by skills and attitudes. These improvements were particularly notable among lower- to mid-level consultants, especially associate consultants. The training highlighted the need to further health advisors' understanding of PE and the opportunity training can provide.

**Conclusions:**

Patient engagement (PE) training significantly improves healthcare consultants' self-perceived knowledge and skills while increasing attitudes and promoting patient-centered approaches throughout the life science industry. This study highlights the importance of standardized PE training programs in enhancing healthcare outcomes and advocates for the integration of patient engagement in healthcare research and development.

**Supplementary Information:**

The online version contains supplementary material available at 10.1186/s40900-025-00711-5.

## Abstract (plain language summary)

In recent years, the healthcare industry has realized how important it is to engage or involve patients when creating healthcare products and doing research. This approach is called patient engagement or involvement. Although there are training courses on how to engage patients throughout the research lifecycle, there are no courses specifically targeted toward health advisors. A healthcare advisor or consultant is a professional who provides guidance to companies conducting projects throughout the product development lifecycle. This article describes the testing of a training course to teach advisors working in the life sciences and healthcare field about patient engagement. The researchers wanted to see if healthcare consultants from a single company with teams in the U.S. and Europe thought their knowledge, skills, and attitudes on patient engagement would improve after taking a course. They gave participants a test to measure their knowledge, skills, and attitudes before and after the course. After the course, the consultants perceived themselves as more knowledgeable, skilled, and positive about including patients in research. Overall, this study shows that a targeted training program, including relevant business cases, can potentially help health advisors improve their ability when engaging patients throughout the product development process.

**Supplementary Information:**

The online version contains supplementary material available at 10.1186/s40900-025-00711-5.

## Background

In the past decade, there has been growing recognition and implementation of patient engagement (PE) throughout the product development lifecycle. This shift is largely due to unified support from stakeholders, including patient advocacy organizations, political and regulatory bodies, policymakers, patient representatives, healthcare providers, and patients [1–4]. Similar to training modules in research methodology strategies, efforts to establish PE training modules can go a long way to ensure PE is properly adopted, executed, measured throughout the healthcare industry, and democratized among relevant stakeholders.

Patient engagement aims to enhance the quality of healthcare by emphasizing patient-centricity, education, and empowerment within patient communities [2]. A study conducted by the European Medicines Agency (EMA) highlights that PE brings to light real-life experiences related to medical conditions and treatments, offering unique patient perspectives and addressing often overlooked concerns [5], such as the emotional burden experienced throughout the patient journey [6]. Understanding these perspectives is valuable for healthcare stakeholders, leading to improved regulatory outcomes, greater research impact, cost-effective development, and strengthened relationships with patient communities [7]. By incorporating patient’s lived experiences, healthcare outcomes can be improved, hospitalizations can be reduced, and the quality and efficiency of health services can be enhanced [8].

Patients provide invaluable insights throughout the product development lifecycle, which includes clinical research design, regulatory processes, market access, and commercialization. Engaging with patients early in the research process aligns studies with real-world needs, thereby enhancing the relevance and impact of new products [3]. Patient input in clinical trials can lead to improved trial design, increased recruitment and retention rates, and enhanced data quality [4]. These enhancements support stronger regulatory submissions, fostering transparent and ethical decision-making [3]. Patients play a crucial role in protocol development, informed consent processes, and regulatory submissions, offering insights into the risks and benefits of treatments [7]. Their experiences significantly influence regulatory decisions, ensuring they reflect patient preferences and contribute to a better understanding of a treatment's real-world impact [9].

In the realm of market access and health technology assessment, involving patients demonstrates the inherent value of therapies from their perspective [10]. Including patients in health economics research, quality of life assessments, and the development of patient-reported outcome measures generate robust evidence. This evidence provides both clinical and economic benefits, which aids reimbursement decisions and ensures patients have access to necessary therapies [7, 10]. Ultimately, engaging patients in commercialization efforts helps to tailor marketing strategies and support programs [11]. By involving patients in market research, branding, and post-marketing surveillance, trust is built, and treatment safety is ensured [8].

### Defining PE

Currently, there is no universally accepted definition of PE; however, various definitions have been proposed by prominent organizations in the field. Examples include The U.S. Food & Drug Administration (FDA) [12], The International Society for Pharmacoeconomics and Outcome Research [13], and The Patients Active in Research and Dialogue for an Improved Generation of Medicine Consortium [3, 4]. These organizations emphasize the importance of meaningful collaboration between patients and healthcare professionals throughout the research and medicine development process. They highlight the value of incorporating patients’ unique insights and experiences, regarding them as essential partners in the journey. This cooperative approach enhances learning, sharpens decision-making, and fosters effective teamwork in healthcare innovation. For the purposes of this article, *we* define PE as *“the systematic approach to ensure that patients’ experiences, perspectives, and priorities are captured and meaningfully incorporated into the whole life cycle for product development and management by establishing a bidirectional partnership between manufacturers and patients. The goal of this approach is to generate products and solutions that serve patients’ needs, increase access and adherence to care, and improve health outcomes”* [14].

PE aims to improve healthcare quality and delivery by prioritizing patient centricity, education, and empowerment within patient communities [2]. According to a study from EMA, PE uncovers information that can surface real-life experiences of conditions and treatment experience, offering patients’ perspectives and raising concerns that are generally not considered [5]. Stakeholders in the healthcare industry involved throughout the product lifecycle can benefit from understanding the patient perspective. This leads to regulatory and access success, increased research impact, cost-effective research and development, knowledge, and better relationships with patient communities [7]. Similarly, incorporating the patient perspective can improve healthcare outcomes, reduce hospitalizations, and enhance the effectiveness, efficiency, and quality of health services as well as quality of life [7].

### Healthcare consulting and its impact on patient engagement

In recent years, healthcare consultants, or consultants, have been hired to handle traditional operational improvements in hospitals, insurance companies, pharmaceutical developers, and medical device manufacturers. This was mainly caused by an advancement in health technology and changes in the regulatory environment [2, 15]. As healthcare standards switch from fee-for-service business models to value-based or patient-attribution payment models, healthcare consultants are expected to empower patients and improve their outcomes, giving them a relevant role in the product lifecycle [2]. To meet this demand, they can guide industry leaders and their companies to become more patient-centric by developing and implementing a PE culture in the organization and among its employees [2].

In a consultancy firm, defined levels of seniority are vital for project success, with individuals typically spending 2 to 3 years at each stage [16]. Entry-level positions typically focus solely on executing and supporting projects through the delivery of content and analysis [16]. As employees advance in their careers by gaining consulting skills, industry knowledge, and professional development education, they become associates. In this role, they define project objectives, engage with clients, and work to enhance their project management and leadership skills with guidance from senior roles [16]. After refining consulting skills and gaining industry knowledge, associates become consultants who manage the execution team and projects, taking direct responsibility for their outcomes [17]. With further advancement, they can attain senior roles such as senior consultant, engagement manager, principal, and partner, where the focus shifts to business growth, innovation, and strategic objectives [2, 16].

### The need for a PE training intervention

To effectively involve patients, all stakeholders advising in the healthcare industry need to grasp how to implement a PE strategy that builds trust and cooperation. It is essential to adopt practices that align with patient values and preferences to improve health outcomes. However, there is still room for improvement in embracing patient-centered practices [3, 4, 18].

**Fig. 1:**
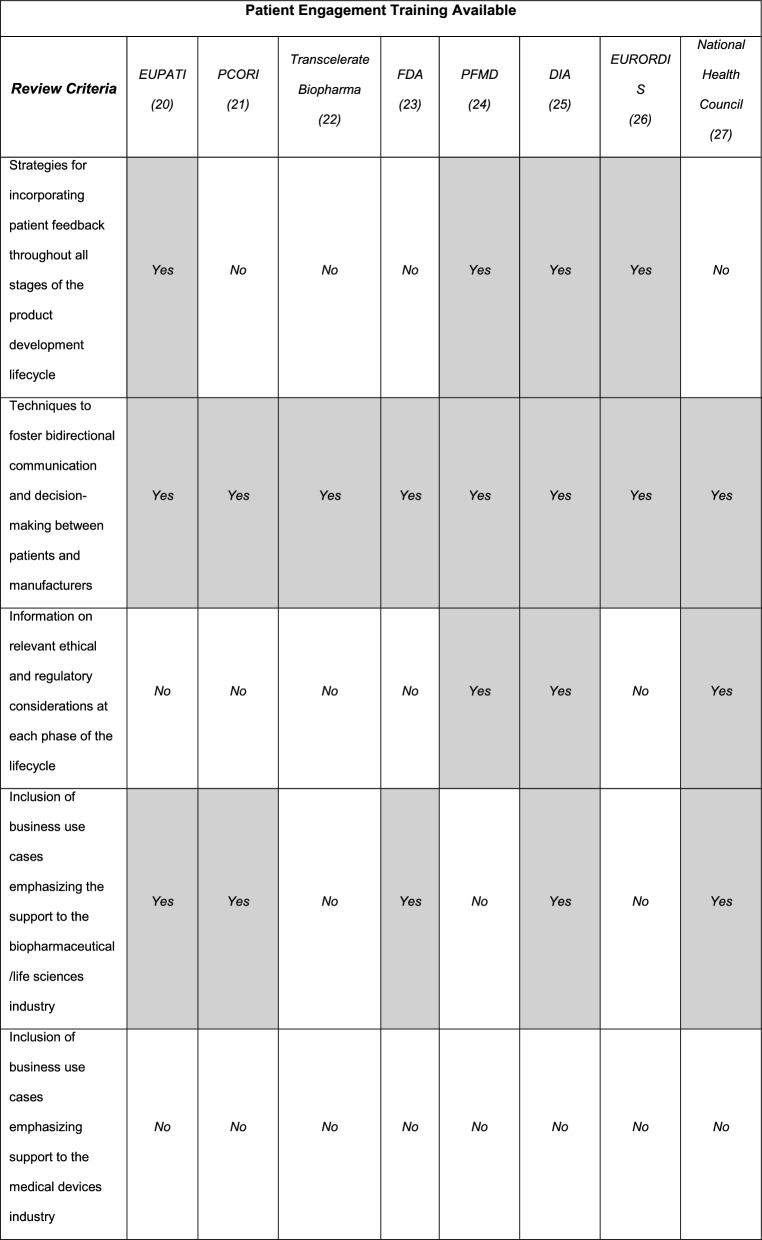
Comparison of available patient engagement training (non-exhaustive list)

Currently, there are inconsistencies across training programs focused on PE, with a gap regarding effective implementation techniques when teaching healthcare consultants. This gap stems from a lack of clarity around what PE entails, particularly concerning how to carry out consulting activities [11, 19]. To tackle this issue, various frameworks and training programs have been introduced to outline best practices in patient engagement. They also describe the foundations and best practices in engagement (Fig. 1).

Existing training courses provide a good overview of tactics, processes, and steps to engage patients in the development of a healthcare solution. However, these courses may fall short when training healthcare consultants working in the life sciences due to the lack of emphasis on the differences in each stage of the healthcare product lifecycle and the lack of business cases applicable to advise the healthcare industry. Addressing this gap may provide healthcare consultants working in the life sciences with the knowledge, skills, and attitudes necessary to provide informed advice to companies working in the healthcare landscape, ensuring solutions are developed considering the patients'input.

The training programs listed above offer valuable foundations of PE from various stakeholder groups from industry and patient organizations. However, there remains a need for a PE training course tailored specifically to healthcare consultants. While the DIA’s PE Certificate Program and PCORI’s patient engagement resources offer insights into PE for various stakeholders, they lack the detailed guidance required to guide healthcare consultants when implementing PE strategies throughout the entire product lifecycle. Healthcare consultants can play an important role in advising industry leaders on becoming patient-centric; however, existing training does not adequately address the unique requirements of this group. Therefore, developing a specialized PE training program for healthcare consultants is essential to fill this gap in the current marketplace and ensure they can effectively foster patient engagement.

In this study, we present a training course on PE tailored to the life sciences industry. Specifically, it is dedicated to personnel who have the responsibility to provide advice to companies and clients developing healthcare solutions across the product development lifecycle. This course is designed around real-world business cases to help healthcare consultants effectively integrate PE into their work. Additionally, we aim to address the following research questions: (1) Is there a significant difference in the attitudes, knowledge, and skills of healthcare consultants toward PE after completing a consultant-focused PE training course? (2) Does the consulting seniority level influence changes in perceived attitudes, knowledge, or skills related to PE following the PE training course? (3) As a result of the training, is there any significant association between attitudes, knowledge, and skills in subscale outcome scores?

## Methods

### Study design

The aim of this study was to assess whether a patient engagement (PE) training course led to a positive shift in healthcare consultants’ self-perceived knowledge, skills, and attitudes (KSAs) related to engaging patients throughout the product development lifecycle. To evaluate this, a prospective one-group pretest–posttest quasi-experimental design was employed, comparing KSA scores before and after completion of the training.

The target population comprised English-speaking healthcare consultants employed in the consulting division of a healthcare and life sciences advisory firm called Alira Health^©^. Employees were invited to participate in the study through company-wide newsletters outlining the study, with an attached screening questionnaire to be used for determining eligibility. Recruitment materials clearly stated that participation was optional. Employees who met the inclusion criteria were included in the study: (1) Employed at the seniority level of associate, consultant, senior consultant, engagement manager, principal, or partner; (2) working within the consulting business unit; and were (3) able to read and willing to sign the consent form. Exclusion criteria included (1) current or recent (30 days) enrollment in a research study; (2) participation in the creation of the patient engagement training program; and (3) being a member of the patient engagement division in the company. A total of 101 employees signed and returned the consent forms and met the eligibility criteria. To reduce dropout rates, the study provided participants with flexible timelines to fit their schedules, sent reminders every two weeks, and offered tailored assistance to help overcome study-related barriers to completion. Participants who failed to complete both the pre-test and post-test were excluded from the final analysis. Due to our study's pre-test and post-test design, direct comparisons between time points were essential, which rendered incomplete data unsuitable for analysis. Of these, 80 healthcare consultants specializing in various health disciplines and products located throughout the United States (Boston area) and several European countries (Spain, the United Kingdom, Switzerland, Italy, and France) were included and completed all study activities within the designated 2-month period. Participants completed three study activities, starting with a pretest (further explained in section"Evidence-based practice questionnaire"), a PE course (described in section"Training course"), and a posttest (using the same tool as in the pretest activity). The only participant characteristic recorded was seniority level.

### Materials

#### Training course

The training course was self-paced, taking approximately 3.5 h to complete, and was made available digitally through the training portal Workday, Inc^©^. For two months, participants could access the course, and completion rates were recorded and reviewed weekly by the study researchers. The training course was developed and evaluated by patient engagement and clinical research experts from Alira Health, including their Chief Patient Officer and patient representatives from the Center for Patient Advocacy Leaders. The aim of the training course is to create a positive shift in healthcare consultants'self-perceived KSAs by educating health advisors on the background of PE, its purpose, and how to incorporate it into consulting projects. To achieve this, the course was designed with business-relevant content included in each module. Each module reflects the goal of incorporating patient feedback to improve product development and management. They include practical exercises, value propositions, business use cases, and regulatory guidance to demonstrate how PE activities can support pharmaceutical and medical device companies, strengthen patient-centric strategies, and ensure products align with patient needs. This collaboration highlights a bidirectional partnership focused on developing solutions that improve health outcomes and promote adherence to care.

**Fig. 2:**
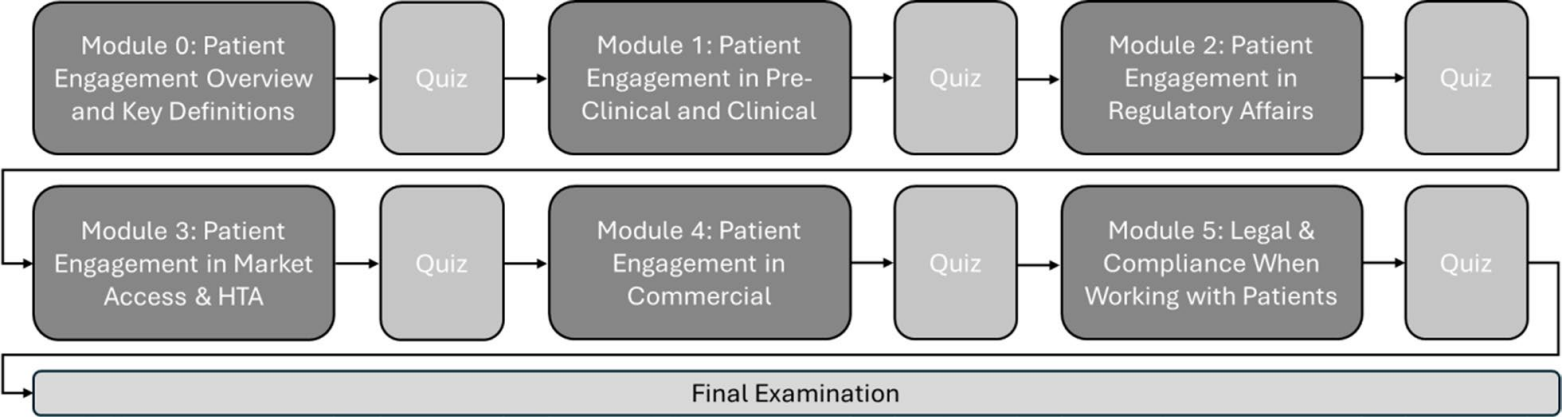
Patient engagement course flow

The training modules were designed to focus on the requirements of healthcare consultants working in the life sciences industry, emphasizing the distinct stages of the product development lifecycle and adding business cases specific to the companies they advise. Through the course, participants learn foundational concepts of patient engagement, supported by concepts of patient engagement from the FDA and EMA. The course covers legal and compliance principles throughout the lifecycle, equipping advisors with the skills to provide informed advice and ensure client solutions are met with substantial patient input (Fig. 1). To assess participant's acquired knowledge, the course incorporated quizzes at the end of each module (Fig. 2). These quizzes included a variety of question formats, such as ordering, matching, and fill-in-the-blank, to encourage retrieval practice, a technique shown to enhance long-term knowledge retention. While quizzes primarily assessed Knowledge (K) as a foundational component, Skills (S), and Attitudes (A) were captured through pre- and post-course self-perception surveys, which demonstrated statistically significant improvements across all three KSA domains.

#### Evidence-based practice questionnaire

Healthcare consultants’ attitudes, knowledge, and skills toward PE were measured before and after the PE course via an adapted version of the Evidence-Based Practice Questionnaire (EBPQ) by Upton & Upton [20], which can be viewed in supplementary materials 1. The EBPQ is a self-report questionnaire that measures the use of evidence-based practices through attitudes, knowledge, and skills. The EBPQ is an appropriate tool to assess self-perceived in KSAs due to its proven reliability and validity in various settings [21]. This method has been widely adopted in studies to measure the effectiveness of educational interventions and training programs, making it a robust choice for evaluating changes in health advisors’ competencies [20, 22]. The original EBPQ developed by Upton and Upton [20] has an internal reliability of 0.87, ranging from 0.79 to 0.91 for each subscale. With permission from the author, the EBPQ was modified to include information on PE principles and practices gathered from the course content. Participants were assessed using an adapted version of the Evidence-Based Practice Questionnaire (EBPQ). While the original EBPQ was developed to assess KSAs in nursing, this study fully modified the items to reflect knowledge, skills, and attitudes specific to Patient Engagement (PE) training for healthcare business consultants. The original instrument's overall structure and scoring method were retained, but the content was adjusted to align with key learning objectives of PE training. The modified questionnaire consists of 18 items measuring attitudes, knowledge, and skills toward PE (Table S1). The internal reliability and consistency of the modified survey for KSA scales were assessed using Cronbach’s alpha. The reliability coefficients were α = 0.87 for the knowledge subscale, α = 0.93 for the skills subscale, and α = 0.80 for the attitude subscale, indicating acceptable to good internal consistency.

Using the modified EBPQ, participants’ self-perceived KSAs in PE were evaluated using the modified version of the EBPQ. Their attitudes were assessed with four questions on a 1 to 7 Likert scale, where higher scores indicate more positive views (maximum 28). Knowledge and skill levels were rated on a 1 to 5 scale, with higher scores reflecting greater knowledge and ability (maximum 35). Details of the adapted EBPQ scale and a summary of the changes to the original scales are available in the supplementary materials (Table S1).

### Data collection and analysis

Upon obtaining approval from the institutional review board, healthcare consultant were invited to participate via email. The invitation included a study description and an eligibility survey. Eligible participants received an invitation email and were asked to complete an electronic informed consent form via DocuSign^©^. Upon confirmation of their consent, they were emailed a pretest questionnaire. After completing the pretest, the participants took part in the PE training course, followed by a post-test questionnaire. The study collected data to assess the effectiveness of the PE training course in enhancing participants'self-perceived competency of knowledge, skills, and abilities (KSAs) related to implementing PE. A one-tail paired samples t-test was used to compare scores before and after the PE training course, evaluating changes in total and KSA subscale scores. The same test was conducted to examine changes in the pre-and post-test KSA subscale scores across the six seniority levels, as detailed in section"The need for a PE training intervention". In addition, the study utilized bivariate Pearson's correlation coefficient to explore potentially significant relationships between the pre-test and post-test KSAs. All analyses were conducted via Microsoft Excel v.16.78, with the significance level set at *p* < 0.05.

Each subscale score was calculated by summing the responses across all items within that subscale. To ensure comparability across subscales, we applied min–max scaling to the attitude’s subscale, adjusting its range from 4–28 to 5–25, aligning it with the scoring range of the knowledge and skills subscales. The total score was then obtained by summing the rescaled subscale scores, providing an overall measure of perceived competency in patient engagement.

## Results

**Fig. 3:**
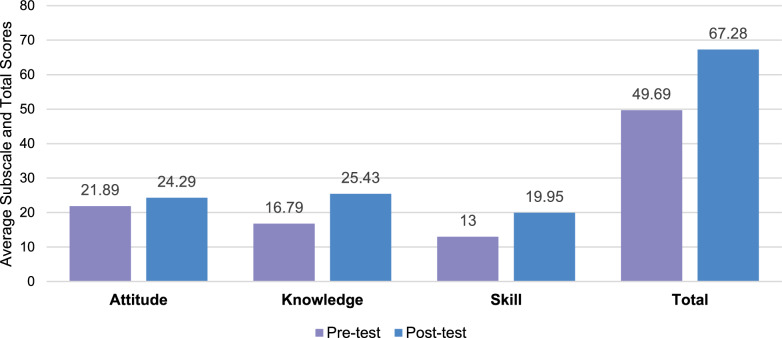
Pre-to-post-test differences in attitudes, knowledge, skills, and total. Note: All results were significant among the subscales and total test scores

Following the study’s end, the pre-test, training course completion, and post-test were completed by 80 participants. A total of 21 employees did not complete the entire training program and were, therefore, removed from the final analysis dataset. Following the training course, most participants rated themselves with higher attitudes, knowledge, or skills following the PE training course for healthcare consultants (Fig. 3). Of those who participated, the majority of participants were associate consultants (32.5%), followed by senior consultants (23.8%), consultants (20%), engagement managers (13.7%), principals (5%) and partners (3.5%). The percentages agree with the typical proportion of these seniority levels within a consultancy firm.

### Impact of a PE training course on self-perceived competency

Overall, healthcare consultants'positive self-perception of PE increased by 35% following the online training course. Data analyses among the KSA subscales revealed that healthcare consultants experienced the most significant improvement in knowledge (51%) and skills (53%) toward PE from the pretest to the post-test (Table S2). Finally, attitudes, although having the smallest increase (10%), showed a statistically significant increase on the post-test (Fig. 3).

### Impact of a PE training course across seniority levels

**Fig. 4:**
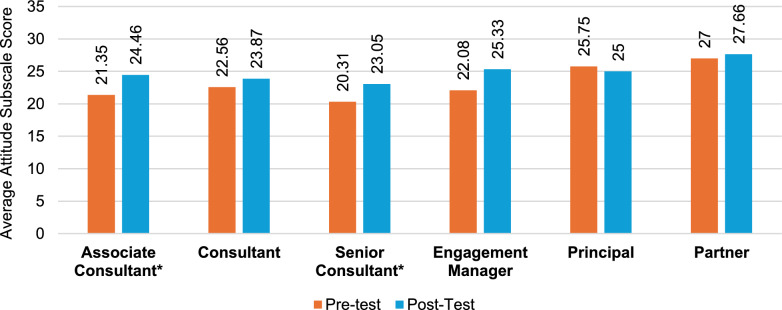
Attitude change by seniority level. Note: An asterisk (*) indicates a significant difference between pretest and posttest scores

**Fig. 5:**
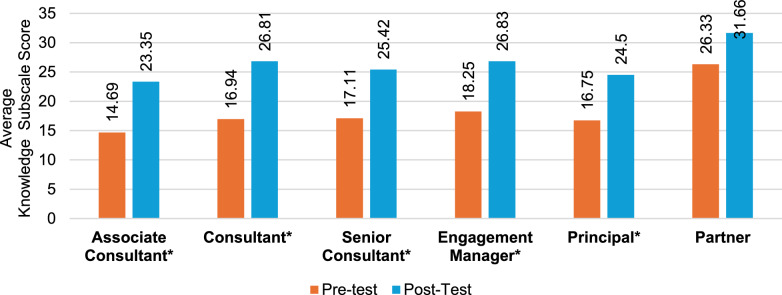
Knowledge change by seniority level. Note: An asterisk (*) indicates a significant difference between pretest and posttest scores

**Fig. 6:**
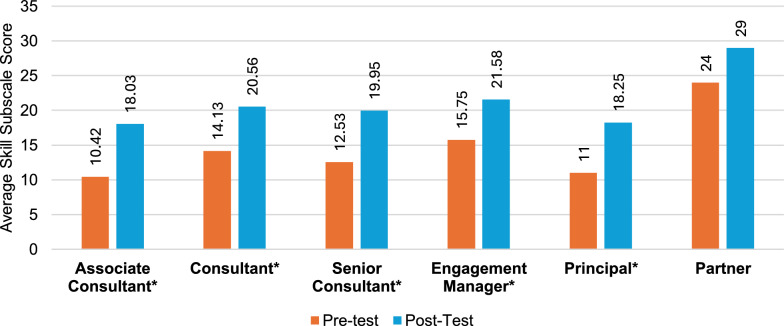
Skill change by seniority level. Note: An asterisk (*) indicates a significant difference between pretest and posttest scores

Following the educational course, the total questionnaire results indicate that all seniority levels, apart from partners, demonstrated marked variations in terms of improved self-perception of KSAs (Fig. 4), with the greatest improvements seen in associate consultants (41%) (Table S3), followed by senior consultants (37%) (Table S5) and engagement managers (32%) (Table S6). The results of knowledge (Fig. 5) revealed the greatest increase in associate consultants (59%), followed by consultants (58%) (Table S4) and senior consultants (49%). The greatest increase overall was observed in associate consultants’ skill subscale scores (73%), followed by principals (65%) (Table S7) and senior consultants (59%) (Fig. 6). Unlike knowledge and skills, attitudes toward PE showed minimal improvements at all seniority levels except for engagement managers (14.7%) and associate consultants (14.5%); however, at the principal and partner levels, scores were already high in the pretraining survey (Tables S7 and S8). Specifically, statistically significant (*p* < 0.05) attitude changes were observed among associate consultants, senior consultants, and engagement managers. More importantly, of these groups, associate consultants (d = 0.81) and engagement managers (d = 0.86) displayed a large effect size, indicating a potential impact on workplace attitudes (Fig. 4). Notably, no considerable attitude changes were observed for consultants, principals, or partners. Associate consultants had the greatest increase in post-test scores among all seniority levels, except for the attitudes scale.

### Relationships between attitude, knowledge, and skill subscales

**Fig. 7:**
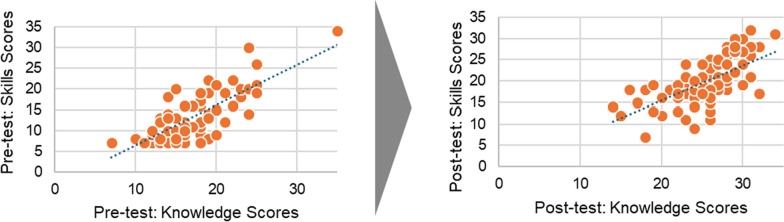
Relationship between knowledge and skills pre- and posttest

As a result of the training, the only strong positive association found was between the total group scores of healthcare consultants'perceived knowledge and skills. This relationship is demonstrated by a high correlation of 0.7 on a scale from −1 to 1, as illustrated in Fig. 7. This relationship became weaker, dropping to 0.6 following the post-test; however, this still indicates a moderate positive relationship between knowledge and skills (Fig. 7). Additionally, the analysis revealed a lack of significant correlation between the skills scores associated with attitudes and those related to knowledge.

## Discussion

The research outcomes highlight the effects of a PE training course on the self-perceived attitudes, knowledge, and skills of healthcare consultants working in one company and across various seniority levels and locations within the U.S. and Europe. All three study activities were completed by 79% of enrolled participants. A challenge to achieving study completion was the timeframe of two months allocated for the execution of all tasks, particularly while participants were simultaneously engaged in their professional responsibilities.

The findings suggest significant improvements in KSAs following training, particularly among lower- to mid-level consultants. The most notable changes were observed in knowledge, followed by skill, indicating that the training course was especially effective in imparting information and concepts related to PE. This is crucial, as a sound understanding of PE is foundational for implementing effective patient-centered principles, practices, and processes.

Similarly, the results revealed a relationship between knowledge and skill scores, which could indicate that those who feel more knowledgeable also perceive their skill as higher following patient engagement training. This practical aspect of the course is vital for translating theoretical knowledge into actionable consulting activities and strategies that can enhance patient care and outcomes. Initial attitudes prior to the PE training course were high, but we observed significant improvements toward greater appreciation and prioritization of PE following the course. Such attitudinal shifts can foster a culture throughout the healthcare industry that values patient perspectives and actively incorporates them into decision-making processes.

### Strengths and limitations

#### Strengths

One of the primary strengths of the PE training course described in this article is that it provides a comprehensive approach to integrating PE throughout the entire product development lifecycle, emphasizing project-based methodologies for effective implementation. While many comparable programs provide extensive insights on applying PE throughout the product development lifecycle, many are tailored to those working within the biopharmaceutical sector [23–26] or are more general to target a wide variety of industry stakeholders [27–29]. In contrast to existing PE training, the proposed training addresses the needs of healthcare consultants by incorporating practical applications of PE across both pharmaceutical and medical device sectors.

Though the FDA [24], PCORI [28], and the National Health Council [30] offer well-regarded insights on best practices, their tools and guidance are not cohesively organized to cover the entire product development lifecycle. Training provided by PFMD [25], EUPATI [27], and DIA [26] addresses implementation across the full lifecycle; however, unlike the proposed PE training, their modules do not follow the chronological sequence of events aligned with the product development process when presenting patient engagement applications.

All examined courses (Fig. 1) provided illustrations and case studies on implementing PE across different phases of the product development lifecycle. In contrast, our proposed course integrates these elements at each stage, with a particular focus on guiding PE activities to ensure effective collaboration and outcomes with clients, primarily focused on ensuring the adequacy and relevance of patient engagement content for healthcare consultants rather than on the specific delivery approach. However, the course structure aligns with two well-established educational theories: Andragogy [31] and Bloom’s Taxonomy [32]. Andragogy emphasizes self-directed learning and the importance of prior experience in adult education, which is reflected in the course’s modular design that allows consultants to engage with content most relevant to their needs [31]. Meanwhile, Bloom’s Taxonomy provides a structured framework for cognitive development, which aligns with the quiz-based assessments used in the course [32]. The quizzes primarily evaluated knowledge acquisition and recall, serving as the foundation for skill development and real-world application in consulting scenarios. While the course was not explicitly built upon these theories, its structure inherently supports principles of progressive learning and self-guided engagement, which are key to both frameworks.

#### Limitations

To the best of our knowledge, this study is the first to measure the effects of a self-paced online training program specific to healthcare consultants within a life sciences company. Our findings demonstrate that the course significantly impacted healthcare consultants'self-perception of their ability to improve overall attitudes, knowledge, and skills related to patient engagement. It assesses the effects of an educational program on healthcare consultants as a whole and, on average, changes in the seniority level post-intervention, helping to identify whether the seniority level impacts scores. Additionally, by examining the correlation between attitudes, knowledge, and skills, we demonstrated a link between increased knowledge and improved skills. While knowledge and skills showed positive correlations in the pre-test (r = 0.6), and post-test (r = 0.7), other subscale associations did not reveal statistically significant correlations. This indicates that although the training improved attitudes, the direct association with knowledge and skills remains uncertain, possibly due to differences in the development of attitudes compared to cognitive and behavioral competencies. Future work is necessary to understand the relationship between attitudes, knowledge, and skills subscales.

A main limitation of the study is the non-randomized design, as the study was conducted within one consulting firm, which cannot be generalized to other companies or countries. Although we observed an improvement in attitudes, it is plausible that those willing to participate in the study may already have had a higher inclination towards patient engagement, potentially influencing the results. Moreover, owing to ethical standards within the company under study, certain demographic information, such as age, educational background, geographic location, and related training experience, was not identified and could lead to a more robust development of when and how education should be implemented.

A important limitation of this study is the attrition of participants. Specifically, 21 employees did not complete the training, leading to their exclusion from the final analysis. Given that the study relied on paired pre-test and post-test data, incomplete responses could not be utilized. This exclusion could create bias, as those who completed the training may systematically differ from those who dropped out (e.g., in terms of motivation or availability), potentially restricting the generalizability of our findings. Additionally, the diminished sample size might have impacted the statistical power of our analysis. Future studies should explore strategies for reducing attrition, such as flexible scheduling, improved engagement measures, or offering partial credit for incomplete participation.

As the survey initially designed to assess healthcare workers was adapted to focus on patient engagement for this study, the interpretation of Cronbach’s alpha should be approached with caution. Although the overall questionnaire score of α = 0.91 and α = 0.93 for the skills subscale indicates strong reliability, it also raises the concern that the items may be overly similar or redundant. Future research should strive to validate these findings with a larger and more diverse sample. Additionally, conducting an item analysis and making necessary revisions would be beneficial. This study has some limitations. First, while the adapted instrument retained the structure and scoring approach of the Evidence-Based Practice Questionnaire (EBPQ), the specific items were fully modified to assess Patient Engagement (PE) competencies. Consequently, this adaptation represents a first step in measuring self-perceived competency improvements related to PE training, rather than a fully validated theoretical model. Similarly, self-reporting is a limitation, as it does not measure actual practice, and changes in attitudes might be overstated due to social desirability bias. Participants may have felt pressured to report improvements in their attitudes and skills due to the positive framing of PE training. This potential bias suggests the need for future studies to incorporate objective measures of skill and knowledge application in real-world settings to validate self-reported data.

The exclusion of entry-level health advisors from the study raises questions about the relevance and generalizability of the findings. Including early career health advisors could provide insights into how professional development training can impact competency in PE over time. Additionally, the pre-posttest design might overestimate the benefits of the training due to factors such as participants discussing the training with peers, leading to a third factor influencing the outcomes. This design does not account for external influences that could affect the results. Implementing a control group in future studies could mitigate this limitation and enhance the validity of the findings.

In our analysis, we categorized participants by job role to explore potential differences, but this approach resulted in small sample sizes for Principals (n = 4) and Partners (n = 3). Consequently, this limitation may have hindered our ability to identify statistically significant changes. It is worth noting that high-level positions are typically less numerous within most business consulting organizations, presenting a challenge during the recruitment of individuals. Future research should consider either increasing the sample sizes within these groups or exploring alternative statistical methods, such as aggregating similar roles or utilizing mixed-effects models. These approaches can effectively accommodate small subgroup sizes while still capturing meaningful differences.

An important takeaway from this study is the need to optimize the educational design to enhance engagement and effectiveness across all participant roles. One potential explanation for the lack of significant change in some subgroups is that the training content may cover already known aspects of patient engagement. For instance, Principals and Partners, who typically have extensive experience in the technical and operational aspects of product development in healthcare. Additionally, since patient engagement is encouraged throughout the study organization, senior’s KSA’s may not be representative of senior positions at other consulting companies. Future iterations of the training could incorporate role-specific content or differentiated instructional strategies to ensure that all participants benefit meaningfully. Additionally, testing the content in different environments (i.e., in-person vs. remote) and incorporating follow-up assessments could help reinforce learning and provide a clearer picture of the long-term impact.

### Implications for future research

The PE training course aimed to address the current gap found in PE training courses, which is the lack of clarity on the role of PE at each stage and business cases to advise implementation throughout the pharmaceutical and medical device industry. Our findings have provided initial evidence that targeted training may have the potential to increase self-perceived competence in applying PE across the product lifecycle development stages. Future research should explore the effects of PE training with larger sample sizes across all seniority levels to ensure comprehensive understanding and applicability, as well as capture the actual implementation of PE into consulting practice. Additionally, future research should focus on identifying the most accurate way to test PE ability or competence in healthcare consultants beyond self-perception. Additionally, research should explore the long-term impacts of PE training on consultants'performance and patient outcomes. Developing and validating PE-specific practice models can offer standardized approaches for wider adoption, promoting consistency and efficacy in PE practices across industries. Understanding the sustainability of training effects and their real-world impact on healthcare delivery will provide valuable insights for refining and enhancing educational interventions.

While the current course structure successfully met its primary objectives, future iterations could further enhance the positive impact on self-perceived KSAs by embedding educational theories more explicitly into the course design. The integration of Andragogy, Bloom’s Taxonomy, or other relevant frameworks could inform not only the content structure but also interactive learning strategies that promote deeper engagement and skill application. Additionally, incorporating active learning approaches, such as discussion forums or reflection exercises, could further reinforce learning retention and facilitate the translation of knowledge into practice.

### Implications in the context of clinical research

The outcomes of this research provide insight into how PE training can benefit teams advising on the design, management, and execution of clinical trials involving patients. Despite increased discussions and focus on patient engagement, academic and industry stakeholders may still prioritize traditional approaches over PE [32].However, educating teams on how to implement PE in the design and conduct of clinical trials could help overcome this barrier. The clinical trial industry is projected to become increasingly complex in the next two to three decades, with artificial intelligence and other non-PE-focused data collection and analysis methods likely to become even more widely utilized [33], a focus on training health advisors on the importance of PE and how it can be applied to their consulting work will become even more essential.

As e-learning continues to be a recognized tool for healthcare professionals, with a recognized benefit alongside traditional in-person learning methods [34], the online PE training program utilized in this study could be widely used across various settings, including clinical-stage companies developing new therapeutics, medical devices, and other technologies requiring clinical trials, as well as healthcare-focused consultants providing input into the clinical trial design and management process. The online nature of the training, combined with the perceived benefits shown by the study results, could result in a program that is well accepted by clinical research professionals across all levels of seniority and geographical locations.

### Implications in the context of regulatory

Since 2000, PE has emerged as a gradual and innovative process recognized by regulators predominantly in the United States and the European Union, with gradual uptake in other countries. The rise of patient input into all aspects of drug and medical device development, including regulatory review, has also impacted medicinal product regulation [35]. Convening diverse stakeholder groups of researchers, academic medicine, industry, patient organizations, health professional societies, think tanks, regulators, and experts has led to a clarifying lexicon of how and where patients can be involved in research goals, objectives, methodology, analysis, authorship, dissemination and elucidating local, regional, and national similarities, differences and barriers in PE in research uptake [36]. An increasing number of regulatory bodies and other agencies are investing resources to develop systematic processes to engage patients and caregivers throughout the research lifecycle [37]. While patients voice their call to action to make research more relevant to their needs, concerns, and issues, regulatory bodies hear stakeholder calls for systematic procedures and best practices to train stakeholders at the research Table [38–40]. Training curricula and practical tools for stakeholders, such as those presented here, have a high potential for embedding PE as a necessary part of an integrated research process. The timing is opportune for training and education materials to enhance the adoption of PE throughout the product development cycle in the life sciences sector.

## Conclusions

Providing healthcare consultants with a framework for integrating patient engagement into their work through an online educational course effectively enhances attitudes toward the involvement of patients across the lifecycle and improves the knowledge and skillset to implement it. This study marks the initial exploration of the potential impact an online, self-paced PE training program can have on healthcare consultants, ultimately benefiting patients.

Notably, early career-level consultants showed the most improvement, highlighting the effectiveness of interventions at early career stages. However, positive effects on more experienced consultants demonstrate the value of PE education across different levels of experience. Moreover, integrating standardized PE training with clearer instruction may enhance healthcare consultants’ awareness in clinical and regulatory settings, creating a patient-centric foundation throughout the product development lifecycle. Moreover, future efforts should focus on refining these programs, establishing standardized practice models, and exploring the long-term impact of implementing PE training in consultancies on patient outcomes.

In conclusion, this study illustrates the value of PE-specific education in enhancing the capabilities of healthcare consultants. By fostering positive attitudes, knowledge, and skills related to patient engagement, such training programs can play a pivotal role in shaping the future of healthcare consulting, ensuring that patient perspectives remain integral to the product development process.

## Supplementary Information

Additional file 1

## Data Availability

The participants in this study signed a consent form that explained the protocol, objectives, and steps of the study; however, they did not provide written consent for their data to be shared publicly. As a result, due to the sensitive nature of the research, supporting data is not available. However, aggregated data is presented in the manuscript and supplementary information files.

## Abbreviations

PE: Patient Engagement
EUPATI: European Patients Academy on Therapeutic Innovation
PCORI: Patient-Centered Outcomes Research Institute
DIA: Drug Information Association
FDA: Food and Drug Administration
EMA: European Medicines Agency
EBPQ: Evidence-Based Practice Questionnaire
KSA/KSAs: Knowledge, skills, and attitudes
