# Supplementary material for: Results of a patient engagement training for health advisors: a study of self-perceived competency enhancements
Source: Res Involv Engagem. 2025 May 23;11:54. doi: 10.1186/s40900-025-00711-5 (PMC12100870; doi:10.1186/s40900-025-00711-5)
Supplement: Supplementary file 1 — Additional file 1 [file 40900_2025_711_MOESM1_ESM.docx]

# Supplementary Materials

**Table S1: Comparison of EBPQ for Nurses to Adapted EBPQ for Patient Engagement**

| **EBPQ Original Section** | **EBPQ Original Item** | **Adapted Section** | **Adapted Item** | **Reason for Adaptation** |
| --- | --- | --- | --- | --- |
| Practice Domain | Formulated a clearly answerable question as the beginning of the process towards filling this gap: | Removed | Removed | The original section does not align with the objective of the present study, which is to assess participants' self-perception before and after an educational course. |
| Practice Domain | Tracked down the relevant evidence once you have formulated the question: | Removed | Removed |  |
| Practice Domain | Critically appraised, against set criteria, any literature you have discovered: | Removed | Removed |  |
| Practice Domain | Integrated the evidence you have found with your expertise: | Removed | Removed |  |
| Practice Domain | Evaluated the outcomes of your practice: | Removed | Removed |  |
| Practice Domain | Shared this information with colleagues: | Removed | Removed |  |
| Attitude Domain | (1—Low Attitude) = My workload is too great for me to keep up with all the new evidence.  (7—High Attitude) = New evidence is so important that I make the time in my work schedule | Attitude Domain | (1—Low Attitude) = Patient-informed insights should only be included if requested by the client.  (7—High Attitude) = Patients must always be included in the decision-making process throughout the project. | Attitude statements were revised to reflect patient engagement in the context of healthcare consulting across the lifecycle. |
| Attitude Domain | (1—Low Attitude) = I resent having my clinical practice questioned.  (7—High Attitude) = I welcome questions on my practice. | Attitude Domain | (1—Low Attitude) = Involving patients is not worth the effort, delay, and related costs.  (7—High Attitude) = Involving patients brings value that justifies the resources needed to do this. |  |
| Attitude Domain | (1—Low Attitude) = Evidence based practice is a waste of time.  (7—High Attitude) = Evidence based practice is fundamental to professional practice. | Attitude Domain | (1—Low Attitude) = I prefer to stick to approaches I know are effective within my area of expertise.  (7—High Attitude) = I am willing to bring value to my area of expertise by learning patient-centric approaches. |  |
| Attitude Domain | (1—Low Attitude) = I stick to tried and trusted methods rather than changing to anything new.  (7—High Attitude) = My practice has changed because of evidence I have found. | Attitude Domain | (1—Low Attitude) = By interviewing healthcare providers, I can robustly define the patient's perspective.  (7—High Attitude) = The perspective of other stakeholders cannot replace the patient's perspective. |  |
| Knowledge and Skills Domain  (1 = Poor, 7 = Best) | Research skills | Knowledge Domain  (1 = None, 5 = Superior) | The main activities to overcoming pains in the commercialization phase, with the goal of achieving a high presentation rate and high likelihood of diagnosis, treatment, or quality of life improvement | Knowledge was separated from skills to capture theoretical concepts related to the product development lifecycle. The Likert scale was adapted to a 5-point format. |
| Knowledge and Skills Domain  (1 = Poor, 7 = Best) | IT skills | Knowledge Domain  (1 = None, 5 = Superior) | The patient engagement activities that can be implemented throughout the product lifecycle |  |
| Knowledge and Skills Domain  (1 = Poor, 7 = Best) | Monitoring and reviewing of practice skills | Knowledge Domain  (1 = None, 5 = Superior) | The differences in the clinical guidelines for patient involvement between the Food and Drug Administration (FDA) and the European Medicines Agency (EMA) |  |
| Knowledge and Skills Domain (1 = Poor, 7 = Best) | Converting your information needs into a research question | Knowledge Domain  (1 = None, 5 = Superior) | The different types of patient categories and the input they can provide throughout the product lifecycle |  |
| Knowledge and Skills Domain  (1 = Poor, 7 = Best) | Awareness of major information types and sources | Knowledge Domain  (1 = None, 5 = Superior) | The Return of Investment (ROI) of a patient-centric clinical study |  |
| Knowledge and Skills Domain  (1 = Poor, 7 = Best) | Ability to identify gaps in your professional practice | Knowledge Domain  (1 = None, 5 = Superior) | The legal principles and requirements when engaging with patients |  |
| Knowledge and Skills Domain  (1 = Poor, 7 = Best) | Knowledge of how to retrieve evidence | Knowledge Domain  (1 = None, 5 = Superior) | The Health Technology Assessment and its different phases |  |
| Knowledge and Skills Domain  (1 = Poor, 7 = Best) | Ability to analyse critically evidence against set standards | Skills Domain  (1 = Novice, 5 = Expert) | Ability to incorporate patient preferences and values into a consulting project | We updated the skills statements to better reflect participants' perceived skill levels in applying patient engagement. We used a reputable 5-point Likert scale to measure skill proficiency. |
| Knowledge and Skills Domain  (1 = Poor, 7 = Best) | Ability to determine how valid (close to the truth) the material is | Skills Domain  (1 = Novice, 5 = Expert) | Ability to identify potential patient engagement activities to be conducted throughout the product lifecycle |  |
| Knowledge and Skills Domain  (1 = Poor, 7 = Best) | Ability to determine how useful (clinically applicable) the material is | Skills Domain  (1 = Novice, 5 = Expert) | Ability to tailor a project scope so it includes activities that collect patients’ input |  |
| Knowledge and Skills Domain  (1 = Poor, 7 = Best) | Ability to apply information to individual cases | Skills Domain | Ability to select the most appropriate type of patient organization to work with depending on the project aim |  |
| Knowledge and Skills Domain  (1 = Poor, 7 = Best) | Sharing of ideas and information with colleagues | (1 = Novice, 5 = Expert) | Ability to define the value created by including patient-centric activities when planning a consulting project or initiative |  |
| Knowledge and Skills Domain  (1 = Poor, 7 = Best) | Dissemination of new ideas about care to colleagues | Skills Domain  (1 = Novice, 5 = Expert) | Ability to recognize the potential gaps in clinical research, real-world solutions, and consulting approaches caused by not including the patient's perspective |  |
| Knowledge and Skills Domain  (1 = Poor, 7 = Best) | Ability to review your own practice | Skills Domain  (1 = Novice, 5 = Expert) | Ability to define Patient Engagement |  |

**Table S2: EBPQ Total, Attitudes, Knowledge, and Skills (N = 80)**

| EBPQ subscales | Pre-test mean (SD) | Post-test mean (SD) | 95% CI [Upper, Lower] | *t* | P-value |
| --- | --- | --- | --- | --- | --- |
| EBPQ total scale score | 49.69 (10.10) | 67.28 (9.06) | [19.40, 15.77] | -19.16 | .001 |
| Attitude subscale score | 21.89 (4.07) | 24.29 (3.34) | [2.87, 1.16] | -5.67 | .001 |
| Knowledge subscale score | 16.79 (4.37) | 25.43 (4.17) | [9.61, 7.67] | -17.64 | .001 |
| Skill subscale score | 13.00 (5.58) | 19.95 (5.29) | [8.05, 5.85] | -12.48 | .001 |

**Table S3. EBPQ Attitudes, Knowledge, and Skills Across Seniority Levels – Associate Consultant (N = 26)**

| EBPQ subscales | Pre-test mean (SD) | Post-test mean (SD) | 95% CI [Upper, Lower] | *t* | P-value |
| --- | --- | --- | --- | --- | --- |
| EBPQ total scale score | 45.69 (7.63) | 66.05 (7.47) | [23.41, 17.32] | -13.50 | 0.001 |
| Attitudes subscale score | 21.35 (3.81) | 24.46 (2.83) | [4.64, 1.59] | -4.11 | 0.001 |
| Knowledge subscale score | 14.69 (3.29) | 23.35 (4.47) | [10.48, 6.83] | -9.56 | 0.001 |
| Skills subscale score | 10.42 (3.98) | 18.04 (4.74) | [9.39, 5.84] | -8.64 | 0.001 |

**Table S4: EBPQ Attitudes, Knowledge, and Skills Across Seniority Levels – Consultant (N = 16)**

| EBPQ subscales | Pre-test mean (SD) | Post-test mean (SD) | 95% CI [Upper, Lower] | *t* | P-value |
| --- | --- | --- | --- | --- | --- |
| EBPQ total scale score | 50.16 (9.90) | 65.31 (10.22) | [19.01, 11.30] | -8.07 | 0.001 |
| Attitudes subscale score | 22.56 (3.43) | 23.88 (3.89) | [3.11, -0.48] | -1.50 | 0.07 |
| Knowledge subscale score | 16.94 (3.72) | 26.81 (3.38) | [9.51, 5.74] | -9.14 | 0.001 |
| Skills subscale score | 14.13 (5.40) | 20.56 (5.20) | [9.20, 3.67] | -4.55 | 0.001 |

**Table S5: EBPQ Attitudes, Knowledge, and Skills Across Seniority Levels – Senior Consultant (N = 19)**

| EBPQ subscales | Pre-test mean (SD) | Post-test mean (SD) | 95% CI [Upper, Lower] | *t* | P-value |
| --- | --- | --- | --- | --- | --- |
| EBPQ total scale score | 52.70 (13.33) | 68.14 (10.43) | [19.69, 11.19] | -7.39 | 0.001 |
| Attitudes subscale score | 20.32 (4.46) | 23.05 (3.94) | [4.58, 0.89] | -3.02 | 0.003 |
| Knowledge subscale score | 17.11 (3.85) | 25.42 (3.60) | [9.11, 4.79] | -8.76 | 0.001 |
| Skills subscale score | 12.53 (4.15) | 19.95 (5.06) | [9.05, 3.37] | -6.25 | 0.001 |

**Table S6: EBPQ Attitudes, Knowledge, and Skills Across Seniority Levels – Engagement Manager (N = 12)**

| EBPQ subscales | Pre-test mean (SD) | Post-test mean (SD) | 95% CI [Upper, Lower] | *t* | P-value |
| --- | --- | --- | --- | --- | --- |
| EBPQ total scale score | 49.65 (6.96) | 70.53 (8.23) | [26.27, 15.48] | -8.52 | 0.001 |
| Attitudes subscale score | 22.08 (3.77) | 25.33 (2.21) | [5.82, 0.68] | -2.78 | .008 |
| Knowledge subscale score | 18.25 (3.59) | 26.83 (3.10) | [11.41, 6.92] | -8.89 | .0001 |
| Skills subscale score | 15.75 (6.26) | 21.58 (4.94) | [12.51, 5.49] | -4.58 | .0001 |

**Table S7: EBPQ Attitudes, Knowledge, and Skills Among Seniority Levels – Principal (N = 4)**

| EBPQ subscales | Pre-test mean (SD) | Post-test mean (SD) | 95% CI [Upper, Lower] | *t* | P-value |
| --- | --- | --- | --- | --- | --- |
| EBPQ total scale score | 55.37 (7.02) | 67.50 (8.20) | [14.76, 9.49] | -11.06 | 0.001 |
| Attitudes subscale score | 25.75 (0.83) | 25 (1.22) | [1.92, -3.42] | 0.67 | 0.27 |
| Knowledge subscale score | 16.75 (3.27) | 24.50 (2.60) | [8.54, 1.46] | -4.01 | .01 |
| Skills subscale score | 11 (0.71) | 18.25 (3.34) | [9.26, 6.24] | -3.59 | .01 |

**Table S8: EBPQ Attitudes, Knowledge, and Skills Among Seniority Levels – Partner (N = 3)**

| EBPQ subscales | Pre-test mean (SD) | Post-test mean (SD) | 95% CI [Upper, Lower] | *t* | P-value |
| --- | --- | --- | --- | --- | --- |
| EBPQ total scale score | 55.50 (6.12) | 69.72 (3.85) | [20.45, 8.00] | -5.93 | 0.01 |
| Attitudes subscale score | 27 (1.41) | 27.67 (0.47) | [3.79, -2.46] | -0.55 | 0.31 |
| Knowledge subscale score | 26.33 (6.60) | 31.67 (2.05) | [11.00, 2.34] | -1.01 | 0.20 |
| Skills subscale score | 24 (7.07) | 29 (1.41) | [10.97, 3.03] | -0.89 | 0.23 |
